# Neonatal stress disrupts the glymphatic system development and increases the susceptibility to Parkinson's disease in later life

**DOI:** 10.1111/cns.14587

**Published:** 2024-02-08

**Authors:** Jian Song, Zhen‐Hua Li, Xin‐Yu Xue, Jing‐Cai Meng, Wen‐Xin Zhu, Shufen Hu, Guang‐Yin Xu, Lin‐Hui Wang

**Affiliations:** ^1^ Department of Physiology and Neurobiology Suzhou Medical College of Soochow University Suzhou China; ^2^ Jiangsu Key Laboratory of Neuropsychiatric Diseases, Institute of Neuroscience Soochow University Suzhou China

**Keywords:** aquaporin‐4, glymphatic system, neonatal stress, Parkinson's disease, α‐Synuclein

## Abstract

**Introduction:**

Neonatal stress disrupts brain development and increases the risk of neurological disorders later in life. However, the impact of neonatal stress on the development of the glymphatic system and susceptibility to Parkinson's disease (PD) remains largely unknown.

**Methods:**

Neonatal maternal deprivation (NMD) was performed on mice for 14 consecutive days to model chronic neonatal stress. Adeno‐associated virus expressing A53T‐α‐synuclein (α‐syn) was injected into the substantia nigra to establish PD model mice. Glymphatic activity was determined using in vivo magnetic resonance imaging, ex vivo fluorescence imaging and microplate assay. The transcription and expression of aquaporin‐4 (AQP4) and other molecules were evaluated by qPCR, western blotting, and immunofluorescence. Animal's responses to NMD and α‐syn overexpression were observed using behavioral tests.

**Results:**

Glymphatic activity was impaired in adult NMD mice. AQP4 polarization and platelet‐derived growth factor B (PDGF‐B) signaling were reduced in the frontal cortex and hippocampus of both young and adult NMD mice. Furthermore, exogenous α‐syn accumulation was increased and PD‐like symptoms were aggravated in adult NMD mice.

**Conclusion:**

The results demonstrated that NMD could disrupt the development of the glymphatic system through PDGF‐B signaling and increase the risk of PD later in life, indicating that alleviating neonatal stress could be beneficial in protecting the glymphatic system and reducing susceptibility to neurodegeneration.

## Graphical abstract

Neonatal maternal deprivation could impair the polarized location of aquaporin‐4 and the glymphatic activity through disrupting platelet‐derived growth factor B signaling and then increase α‐synuclein aggregation and susceptibility to Parkinson's disease in later life.

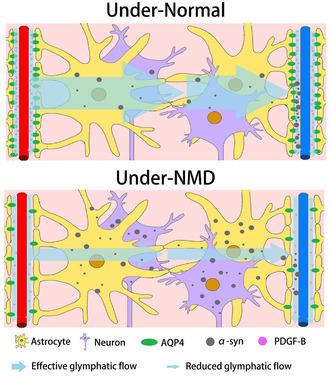

## 1 INTRODUCTION

The glymphatic system is a brain‐wide macroscopic system for the efficient clearance of harmful metabolites such as amyloid β (Aβ) from the brain.^1^, ^2^ The system mediates the flow of cerebrospinal fluid (CSF) into the brain along the peri‐arterial spaces and subsequently into the brain interstitium and then directs the flow towards the peri‐venous spaces, ultimately clearing solutes from the neuropil to the subarachnoid space.^2^, ^3^ The glymphatic flow is mainly facilitated by aquaporin‐4 (AQP4) water channels.^1^, ^4^, ^5^, ^6^ Moreover, the polarized localization of AQP4 in surrounding blood vessels (AQP4 polarization) is critical for glymphatic flow.^7^, ^8^, ^9^ Notably, AQP4 polarization is modulated by the dystrophin‐associated protein complex (DAPC) and extracellular matrix components such as laminin and agrin.^10^, ^11^, ^12^, ^13^

The glymphatic system develops early in life. For instance, the glymphatic flow in rat's brain develop by postnatal day 90 (P90).^14^ Similarly, the glymphatic flow in the mouse brain first emerges in the hippocampus (HPC) at P1 and is established in the cortex (CTX) at P14.^15^ Furthermore, the glymphatic flow depends on AQP4 polarization in the developing brain and a deficiency of platelet‐derived growth factor B (PDGF‐B) disrupts the development of the glymphatic system.^15^ Substantial evidence has shown that exposure to early life stress (ELS), such as maternal neglect, has severe effects on brain development and increases the risk of developing multiple diseases later in life.^16^ These ELS‐related diseases include psychiatric disorders, such as anxiety and depression,^17^ and neurodegenerative disorders, including Alzheimer's disease (AD) and Parkinson's disease (PD).^18^, ^19^, ^20^ However, the potential influence of ELS on glymphatic system development and related diseases remains largely unknown. Recent preclinical studies have indicated that chronic stress in adulthood impairs the AQP4 mediated glymphatic system.^21^, ^22^ These findings inspired us to investigate whether ELS can disrupt glymphatic development.

PD is a prevalent neurodegenerative disease characterized by the degeneration of dopaminergic neurons in the substantia nigra (SN) and the presence of Lewy bodies.^23^ Several studies have implicated the gradual accumulation of misfolded α‐synuclein (α‐syn) in the pathogenesis of PD.^24^, ^25^ Thus, identifying the potential pathways responsible for α‐syn clearance is vital for PD treatment. Previous studies have reported that intracellular α‐syn is degraded through the ubiquitin–proteasome system and autophagy–lysosomal pathway.^26^ Recent pieces of evidence suggested that the glymphatic system is involved in the clearance of extracellular α‐syn.^6^, ^27^ The etiology of PD is related to the interaction between genetic mutations and environmental factors such as stress.^28^ Multiple studies found that neonatal maternal deprivation (NMD), a well‐established ELS model, caused the damage of dopaminergic neurons and aggravated the PD‐like symptoms.^19^, ^29^, ^30^, ^31^, ^32^ However, the underlying mechanisms are not well‐understood. Herein, we hypothesized that NMD might impair glymphatic clearance of α‐syn and accelerate α‐syn accumulation and PD‐like symptoms.

To test this hypothesis, newborn mice were subjected to chronic NMD for 14 consecutive days. An adeno‐associated virus expressing A53T‐α‐syn (AAV‐A53T) was injected into the SN of mice to model PD. Glymphatic activity was determined using dynamic contrast‐enhanced magnetic resonance imaging (DCE‐MRI), fluorescence imaging and microplate assay. Expression of AQP4 and other related proteins in the mouse brain was evaluated using western blotting (WB), quantitative polymerase chain reaction (qPCR) and immunofluorescence (IF). Moreover, the animals' responses to NMD and α‐syn overexpression were observed using behavioral tests. This study suggests that NMD can disrupt glymphatic development, impair α‐syn clearance from the brain, and ultimately aggravate susceptibility to PD later in life.

## 2 MATERIALS AND METHODS

### 2.1 Animals

Adult male and female C57BL/6 mice (2–3 months old, breeders), obtained from the Shanghai Laboratory Animal Center, were housed in cages for mating. The pregnant mice were housed individually until delivery. All pups were housed with the dam until weaning. Animals were maintained in standard housing conditions (room temperature 18–22°C, humidity 30%–50%, light/dark cycle of 12 h). Mice were anesthetized using a combination of xylazine (10 mg/kg; Sigma–Aldrich) and ketamine (100 mg/kg; Li'An Medicine) by intraperitoneal injection. Only males were used in the study, and efforts were made to minimize animal suffering and reduce the number of animals used. All experimental procedures involving animals were approved by the Committee on Animal Resources of Soochow University.

### 2.2 Materials

Fluorescein‐conjugated dextran (D3306, molecular weight: 3 kDa, Dex‐3) was purchased from Invitrogen. Gd‐DPTA (molecular weight: 938 Da) was obtained from CONSUN. HiLyte Fluor‐488‐labeled recombinant human α‐syn (AS‐55457, molecular weight: 14 kDa HiLyte‐488‐α‐syn) was purchased from Anaspec. Adeno‐associated virus expressing A53T‐α‐syn (AAV‐A53T) was designed by OBio Technology. Primary antibody anti‐AQP4 (rabbit, AB3594), anti‐α‐syn (mouse, 36–008; Syn211), and anti‐tyrosine hydroxylase (rabbit, AB152; anti‐TH) were purchased from Millipore. Anti‐PDGF‐B (rabbit, DF6328) was purchased from Affinity Biosciences, and anti‐PDGFRb (mouse, sc‐374573) was purchased from Santa Cruz Biotechnology. β‐actin (mouse, ab008‐100), horseradish peroxidase‐conjugated anti‐rabbit and anti‐mouse IgG (70‐gam007, 70‐gar007) were purchased from Multisciences. Secondary antibodies, including Cy3‐conjugated donkey anti‐rabbit and Cy5‐conjugated donkey anti‐rabbit, were purchased from Jackson ImmunoResearch. DNaseI and 4′,6‐diamidine‐2′‐phenylindole dihydrochloride (DAPI) were purchased from Sigma‐Aldrich. RNAiso Plus (9109), PrimeScrip RT Master Mix (RR036A) and SYBR Premix Ex Taq II (RR820A) were purchased from TaKaRa.

### 2.3 The NMD protocol and experimental groups

**FIGURE 1:**
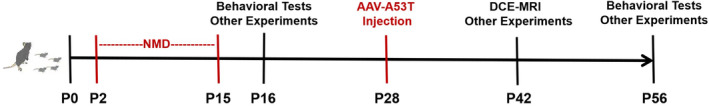
A flowchart of the experiment. Neonatal mice underwent maternal deprivation for 14 consecutive days (P2–15). When NMD and CON mice reached P16 and P42, behavioral changes and plasma CORT were measured to determine the success of the NMD model. At P42, glymphatic activity of both NMD and CON mice were measured using DEM‐MRI, fluorescence imaging, and microplate assay, and clearance of HiLyte‐488‐α‐syn in mice brain was measured using fluorescence imaging and microplate assay following intranigral injection. At P16 and P42, expression of AQP4 and related proteins in mice brain were measured using IF, WB, and qPCR. At P28, AAV‐A53T was bilaterally injected into the SN of some NMD and CON mice. At P56, behavioral changes and expression of human α‐syn and TH of both CON‐A53T and NMD‐A53T mice were measured.

The NMD was performed on mice as previously described.^33^, ^34^ Briefly, pups for the NMD group were removed from the maternity cages and placed in the isolation box with an electric blanket to maintain their warmth (30°C ± 2°C) for 3 h per day, from P2 to P15. After separation, pups were returned to their maternity cages. Pups in the control (CON) group were maintained in maternity cages with a dam and were not exposed to handling. The sex of pups was determined on P22. Female pups were culled and male pups were weaned and housed until use. Only male mice were used mainly because males are more vulnerable to NMD than females according to previous studies.^35^, ^36^ Figure 1 shows a flow chart of the experiment. At P16, behavioral changes, plasma corticosterone (CORT), expression of AQP4 and related proteins were measured in both NMD and CON mice. NMD model was successfully established if mouse displayed depressive symptoms (i.e. increased immobility time in tail suspension test (TST), reduced traveled distance in Open field test). Mice were culled if no depressive symptoms occurred. At P42, glymphatic activity, α‐syn clearance, behavioral changes, plasma CORT, expression of AQP4 and related proteins were measured. At P28, AAV‐A53T was injected into the SN of some NMD and CON mice. At P56, behavioral changes and expression of A53T‐α‐syn and TH of both CON‐A53T and NMD‐A53T mice were measured.

### 2.4 Tail suspension test

As previously described,^21^ each mouse was hung upside down by its tail at a height of 12–16 cm from the ground using a piece of adhesive tape wrapped around the tail 2 cm from the tip. Animals were considered immobile only if they remained motionless. The immobility duration was recorded for 6 min.

### 2.5 Forced‐swimming test (FST)

As previously described,^21^ each mouse was placed into a glass beaker (volume 2 L, 20 × 20 cm) with water depth of 10 cm (25 ± 1°C). Immobility was defined as either the complete cessation of swimming or only minimal limb movement. The total testing period was 6 min. The immobility time was recorded during the last 4 min, which was followed by 2‐min of habituation.

### 2.6 Open field test

As previously described,^21^ mice were placed in the central grid of an open field box (52.5 × 52.5 × 41.5 cm, with nine grids) and monitored by video to observe spontaneous locomotor activity. Animal behavior was evaluated for 10 min followed by 1‐min of adaptation. The total travel distance and time spent in the central area were used for the analysis. After each session, the cages were cleaned with ethanol.

### 2.7 Rotarod test

As previously described,^6^ the mice were placed on a rod (Rotarod YLS‐4C; Yi Yan Science and Technology Development) rotating at a constant speed of 20 rpm for training. The mice were trained for 5 min each time, 3 times per day for 3 consecutive days. In the formal test, each mouse was placed on a rod rotating at a constant accelerated speed (0.1 rpm/s, from 0 to 40 rpm). The latency to fall was recorded for the analysis. The maximum latency was 300 s, and a maximum latency of 300 s was recorded if the mouse stayed on the rotating rod for more than 300 s. The formal test was performed three times for each mouse, and the average was calculated. An interval (at least 30 min) between the two trials was allowed to prevent exhaustion.

### 2.8 Olfactory sensitivity testing

As previously described,^6^ multiple scents with peanut butter (JUMEX) at the dilution of 10^−1^, 10^−2^, 10^−3^, 10^−4^, and 0 were prepared. Prior to testing, each mouse was habituated to a clean cage without bedding for 15 min. The scent was placed in the cage at the opposite end of the animal's current position. The total time spent on the scent (within 180 s) was recorded for analysis, and multiple scents from high to low concentrations were tested. The cages were cleaned with ethanol before the next subject was placed in them.

### 2.9 Estimation of plasma corticosterone (CORT)

Blood samples were obtained from anesthetized mice by heart puncture using a syringe containing a 1% heparin sodium solution. After 15 min of centrifugation at 2000 × g at 4°C, plasma samples were stored at −80°C for use. Plasma CORT concentration was measured using an enzyme immunoassay (EIA) kit following the manufacturer's instructions.

### 2.10 Intracisternal injection

Gd‐DPTA was diluted in phosphate‐buffered saline (PBS) to a final concentration of 4.69%. Dex‐3 was diluted in the artificial CSF (containing 7.247 g of NaCl, 2.184 g of NaHCO_3_, 0.224 g of KCl, 0.493 g of MgSO_4_, 0.193 g of NaH_2_PO_4_, 0.222 g of CaCl_2_, and 1.982 g of C_6_H_12_O_6_, for a volume of 1000 mL) to a final concentration of 0.5%. Intracisternal injection was performed as described previously.^37^, ^38^, ^39^ Briefly, anesthetized mice were fixed in a stereotaxic frame, and the posterior atlanto‐occipital membrane covering the cistern magna (CM) was carefully exposed. The CM was cannulated using a 30G needle attached via PE‐10 polyethylene catheter to a Hamilton syringe. Gd‐DPTA or Dex‐3 (10 μL total volume) was infused using a syringe pump at a flow rate of 1 μL/min for 10 min. Body temperature of mice was maintained at 37 ± 0.5°C with a heating pad, and deep anesthesia was sustained throughout the injection.

### 2.11 Intranigral injection

According to our previous study,^6^ the anesthetized mice were stereotactically administered AAV‐A53T at a concentration of 7.15 × 10^12^ genomic particles (gp)/mL bilaterally into the SN with a microinjector at a flow rate of 0.5 μL/min (1.5 μL total volume). In another experiment, HiLyte‐488‐α‐syn diluted in PBS (0.1%, 1 μL total volume) was injected into the right SN at 0.25 μL/min for 4 min. According to the mouse brain atlas of Paxinos and Franklin (Second Edition, 2001), the coordinates from the bregma were as follows: anteroposterior −3.0 mm, mediolateral ±1.25 mm, and dorsoventral 4.5 mm from the brain surface.

### 2.12 Dynamic contrast‐enhanced magnetic resonance imaging (DCE‐MRI)

**FIGURE 2:**
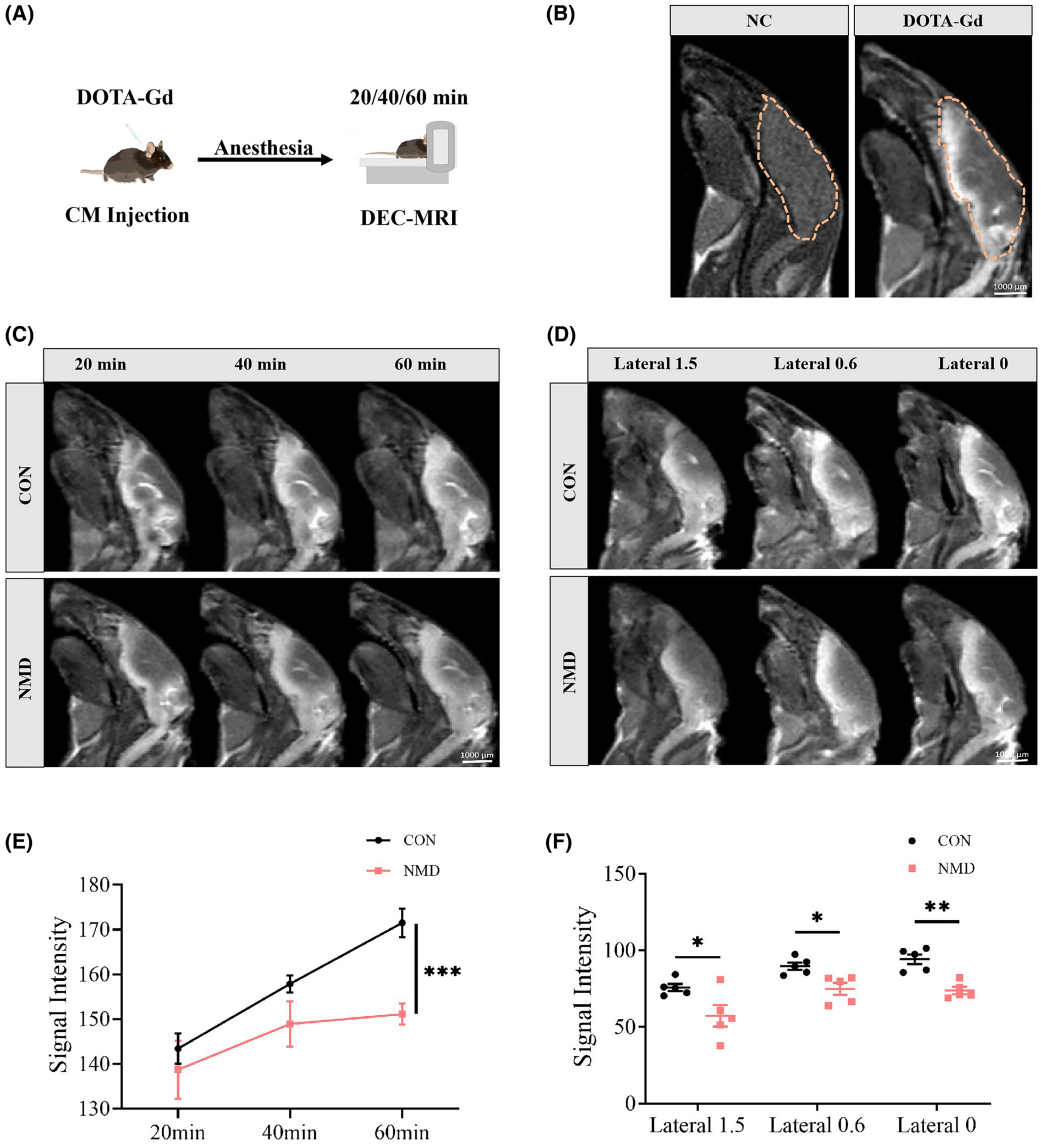
DCE‐MRI showing reduced glymphatic activity in adult NMD mice. (A) Schematic diagram of Gd‐DPTA injection and MRI. (B) Representative sagittal T1‐weighted images (Lateral 0 mm) acquired by MRI 20 min after DOTA‐Gd injection showing that the brain parenchyma, especially the ventral brain, was highly enhanced by contrast, whereas no signal was identified in non‐injected control (NC) mice. The brown boxes depict the brains of mice. (C) Serial images (Lateral 0 mm) displaying the transport of Gd‐DPTA into the brains of NMD and CON mice 20, 40, 60 min following injection. (D) Representative images demonstrating the penetration of Gd‐DPTA into the brains of NMD and CON mice at three slicing regions (Lateral 1.5, 0.6 and 0 mm) 60 min following injection. (E) Quantification of T1 weighted signal in the brain normalized to baseline at three time‐points. (F) Quantification data showing the difference of Gd‐DPTA in the brain at 3 slicing regions between NMD and CON mice 60 min following injection. *n* = 5, *p < 0.05, **p < 0.01, ***p < 0.001, and unpaired‐sample *t*‐test.

CSF circulation was observed using DCE‐MRI as described previously.^5^ Mice were kept under general anesthesia during the experiment, and their body temperature was maintained at 37 ± 0.5°C. Imaging was performed using a Philips 3 T scanner equipped with a 20 mm I.D. quadrature RF volume‐coil. Consecutive T1 weighted 3D FLASH images were obtained at 20 min intervals for a total of 60 min. Intracisternal Gd‐DPTA injection was initiated after the first image (baseline image) was acquired. If no elevation in the signal was detectable in the mouse brain within the first 20 min, the animal was excluded from further analysis. Mice with movement artifacts during the imaging process were also excluded from the study. Regions of interest (ROIs) were drawn on a sagittal image slice that closely resembled the one depicted in Figure 2B, and the mean intensity was determined using the ImageJ software (NIH).

### 2.13 Ex vivo fluorescence imaging

Mice were transcardially perfused with PBS and paraformaldehyde (PFA, 4%) 30 or 90 min after Dex‐3 infusion, or 60 min after intranigral injection with HiLyte‐488‐α‐syn. Mouse brains were gently removed and post‐fixed in 4% PFA overnight at 4°C. For mice that received Dex‐3 infusion, the left brain was sliced into 100 μm coronal sections on a vibratome for quantification on tracer distribution, and the right brain (RB) was sliced into 100 μm sagittal sections to assess the success of the injection. According to our previous study, sagittal brain sections are ideal for assessing the success of intracisternal injection. Large amounts of tracer would be found in the brainstem if injecting into the tissue incautiously. In contrast, if leaky injection occurred, the signal of tracer in the brainstem would markedly reduce in comparison with successful injection.^40^ In doing so, the number of animals used was also reduced. For mice that received HiLyte‐488‐α‐syn injection, the whole brain was sliced into 100 μm coronal sections. The distribution of the fluorescent tracers within the coronal brain slices was imaged and analyzed using a fluorescence microscope (Eclipse TE 2000‐U, Nikon). Whole‐slice montages were integrated using the Virtual Slice module of the Kolor Autopano Giga (V4.4). The distribution of the fluorescent tracer was quantified as previously described.^37^, ^38^, ^39^ Briefly, the fluorescence channels were split and ROIs were defined for each slice. The coverage area of the tracer was quantified using ImageJ.

### 2.14 Tracer assays

The whole RB of the mice and the CTX and HPC of the left brain were dissected 30 or 90 min after the intracisternal injection with Dex‐3. The ipsilateral subcortical region (SCR) of the mice was dissected 60 min after the intranigral injection of HiLyte‐488‐α‐syn. Quantitative measurements of tracer content were performed as described previously.^6^, ^40^ Briefly, brain tissues were homogenized on ice with a corresponding volume of lysis buffer according to their weights (RB, CTX, and SCR: 200 mg/mL, HPC: 100 mg/mL). Homogenized samples were centrifuged at 12,000 *g* at 4°C for 20 min. Supernatants with the volume of 100 μL were added into microplate containers, and the intensity of tracers was spectrophotofluorometrically analyzed at an excitation wavelength (488 nm for Dex‐3, 490 nm for HiLyte‐488‐α‐syn) and emission wavelength (535 nm for Dex‐3, 525 nm for HiLyte‐488‐α‐syn) using a microplate reader (Synergy NEO, BioTek). The contents of Dex‐3 and HiLyte‐488‐α‐syn were quantified from their standard curves, respectively, and expressed as ng/mg of tissue.

### 2.15 Immunofluorescence (IF)

Brain tissues were fixed in 4% PFA and then cut into 100‐μm slices on a vibratome. IF was performed as previously described.^6^, ^21^ Briefly, the free‐floating slices were permeabilized and blocked in PBS consisting of normal donkey serum (5%) and Triton X‐100 (0.5%) for 2 h at 25°C. The slices were then incubated with specific primary antibody (1:500) overnight at 4°C. After washing with PBS, the sections were incubated with fluorescent secondary antibody (1:500) for 2 h at 25°C. The slices were washed in PBS with DAPI (1:1000) for 20 min, washed again with PBS, and mounted. The primary antibodies used were rabbit anti‐AQP4, mouse anti‐α‐syn, and rabbit anti‐TH. The secondary antibodies were Cy3‐conjugated donkey anti‐rabbit and Cy5‐conjugated donkey anti‐mouse. IF was performed using a confocal scanning microscope (FV1200, Olympus).

AQP4 polarization was measured as previously described.^6^, ^10^, ^21^ Briefly, the pixel intensity of AQP4 staining on the perivascular endfeet and parenchyma domains was measured using ImageJ. The ratio of pixel intensity (perivascular area/parenchymal domains) was calculated to represent the degree of AQP4 polarization. In each slice, approximately 10 perivascular areas and 10 parenchymal domains were randomly selected. Approximately five slices per animal were measured in this manner.

### 2.16 Western blotting

After dissection, brain tissues including the HPC, frontal cortex (FC), SN, and striatum (STR) were immediately frozen in liquid nitrogen and then stored at −80°C for use. WB was performed as described previously.^6^, ^21^ Briefly, the immunoblots were performed using 50 μg of total protein extract separated on 10% gels and then transferred electrophoretically to polyvinylidene difluoride membranes. Membranes were blocked with 5% non‐fat milk in Tris‐buffered saline containing 0.1% Tween 20 for 2 h and then incubated with the primary antibody at 4°C overnight. The following antibodies were used: rabbit anti‐AQP4 (1:1000), rabbit anti‐PDGF‐B (1:1000), mouse anti‐PDGFRb (1:100), mouse anti‐α‐syn (1:1000), p‐α‐syn (1:1000), and rabbit anti‐TH (1:1000). After washing, the membranes were incubated with horseradish peroxidase‐conjugated secondary antibodies (1:5000) for 1 h at room temperature. Immunoreactivity was detected using chemiluminescence and visualized by autoradiography (Bio‐Rad). Densitometry calculations were performed using Image Lab 3.0 and ImageJ.

### 2.17 Real‐time PCR

Total RNAs was extracted from the HPC and FC using RNAiso Plus. cDNA was synthesized from total RNA using the PrimeScript RT Master Mix. qRT‐PCR was performed using a 7500 Real‐Time PCR System (Applied Biosystems). Each cDNA sample was amplified in a 20 μL volume with 500 nM final concentrations of each primer, using the SYBR Premix Ex Taq II. The amplification cycles initiated with a denaturing cycle at 95°C for 30 s, followed by 40 cycles of 10 s at 95°C and 30 s at 60°C. The RNA quantities were normalized to β‐actin, and the relative quantification of the target gene was analyzed using the 2^−△△Ct^ method as described previously.^41^ The primer sequences used for qPCR are listed in Table 1.

**TABLE 1:** Primer sequence for real‐time PCR.

Gene	Sequence	
AQP4	Forward primer	TCAGCATCGCTAAGTCCGTC
	Reverse primer	CGTGGTGACTCCCAATCCTC
Laminin	Forward primer	GATAACAGCCACCTACCAGCC
	Reverse primer	ACTGCACTTGTGAGCCCCTG
Agrin	Forward primer	TGGCTACTTCTACGTTGGGC
	Reverse primer	CGCTGTAGCTCACACTCGTT
β‐Actin	Forward primer	GCGGGCGACGATGCT
	Reverse primer	TCATCTTTTCACGGTTGGCCT

### 2.18 Statistical analyses

Prism 7 software (GraphPad) were used for the statistical analysis. The normality of the data was examined using the Shapiro–Wilk test. An unpaired sample Student's *t*‐test was used to compare the two groups (CON vs. NMD). In addition, nonparametric test was used if necessary. All data are presented as the mean ± SEM, and *p* < 0.05 was considered statistically significant.

## 3 RESULTS

### 3.1 NMD impaired the glymphatic activity in adult mice

We first used in vivo DCE‐MRI to investigate whether glymphatic activity is impaired in the brains of adult NMD mice. Serial T1‐weighted imaging at 20 min intervals was performed following intracisternal injection of DOTA‐Gd (Figure 2A). Twenty minutes after injection, the DOTA‐Gd signal in the brain was enhanced, particularly in regions along the ventral brain (Figure 2B). Sequential MRI revealed that DOTA‐Gd progressively entered the brains of both the CON and NMD mice from 20 to 60 min after injection (Figure 2C). Quantification data showed that until 60 min, the DOTA‐Gd signal within the brains of NMD mice remained consistently lower than those of CON mice (Figure 2E). The contrast signal was significantly reduced in the brains of NMD mice than in those of CON mice at three slicing regions, including lateral 1.5, 0.6, and 0 mm, 60 min following injection (Figure 2D,F). These results indicated that glymphatic influx was impaired in adult NMD mice.

**FIGURE 3:**
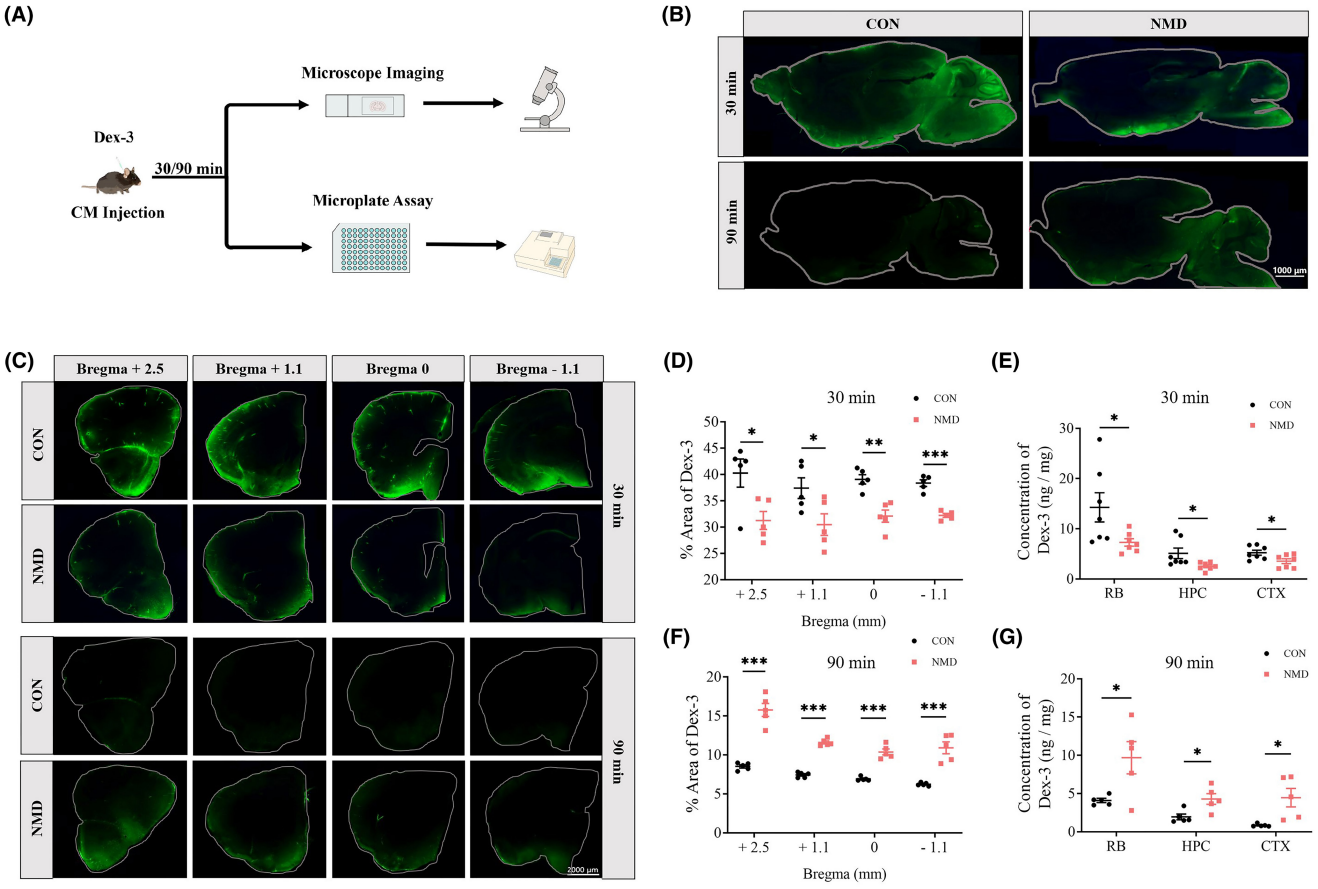
Fluorescence imaging and microplate assay showing the damaged glymphatic activity in adult NMD mice. (A) Schematic diagram of Dex‐3 injection and detection. (B) Representative images of sagittal sections (right brain, RB) demonstrating the distribution of Dex‐3 (green) in the brains of NMD and CON mice at Lateral 0.3 mm 30 and 90 min following injection. (C) Representative images of half coronal sections (left brain) demonstrating the distribution of Dex‐3 (green) in the brain of NMD and CON mice at four slicing regions (Bregma +2.5, +1.1, 0, −1.1 mm). (D, F) Quantification data showing the coverage percentage of Dex‐3 in the half coronal sections at 4 slicing regions 30 min (D, *n* = 5 per group) and 90 min (F, *n* = 5 per group) following injection. (E, G) Microplate assay showing the content of Dex‐3 in the whole RB, left hippocampus (HPC) and left cerebral cortex (CTX) of NMD and CON mice 30 min (E, *n* = 7 per group) and 90 min (G, *n* = 5 per group) following injection. *p < 0.05, **p < 0.01, ***p < 0.001, and unpaired‐sample *t*‐test.

Next, we validated glymphatic influx dysfunction induced by NMD exposure using ex vivo imaging of a brain‐wide fluorescent tracer (Figure 3A). Thirty minutes after the start of intracisternal Dex‐3 infusion, the mice were perfusion‐fixed. Coronal (left brain) and sagittal (RB) sections were vibrated. Representative images showed the distribution of Dex‐3 (green) in sagittal (Figure 3B) and coronal sections (Figure 3C) in both groups. Quantification of the coronal sections showed that the penetration of Dex‐3 was dramatically decreased in the brains of NMD mice than in those of CON mice at Bregma +2.5, +1.1, 0, and − 1.1 mm (Figure 3D).

Furthermore, we used microplate assays to quantitatively determine glymphatic activity (Figure 3A). Mice were decapitated 30 min after intracisternal Dex‐3 injection. Specific brain regions including the RB, HPC, and CTX of the left brain were dissected. These tissues were homogenized in lysis buffer, and the content of Dex‐3 were analyzed using a fluorescence microplate. The result showed that the content of Dex‐3 in RB, HPC and CTX was dramatically lower in the NMD mice than in the CON mice (Figure 3E). These results revealed that the uptake of Dex‐3 by each defined brain region was dramatically decreased in the NMD group than in the CON group, which was consistent with observations made via the imaging approach.

To determine whether glymphatic efflux was impaired in adult NMD mice, we assessed the retention of Dex‐3 in the brain 90 min after intracisternal injection using both ex vivo imaging and microplate assays (Figure 3A). Representative images of sagittal and coronal sections demonstrated that the Dex‐3 signal (green) was extremely weak in the brains of the CON mice at this time point. In contrast, the NMD mice exhibited higher fluorescence levels in the brain (Figure 3B,C). Quantification data showed that at four slicing regions, including Bregma +2.5, +1.1, 0, and −1.1 mm, the residual Dex‐3 in NMD mice was stronger than that in the CON group (Figure 3F). Consistently, the results from the microplate assays showed that the content of Dex‐3 within the RB, HPC, and CTX was dramatically increased in the NMD group than in the CON group (Figure 3G). These results indicated that glymphatic efflux was inhibited in adult NMD mice.

### 3.2 NMD reduced the AQP4 polarization and PDGF‐B signaling in the mice brain

**FIGURE 4:**
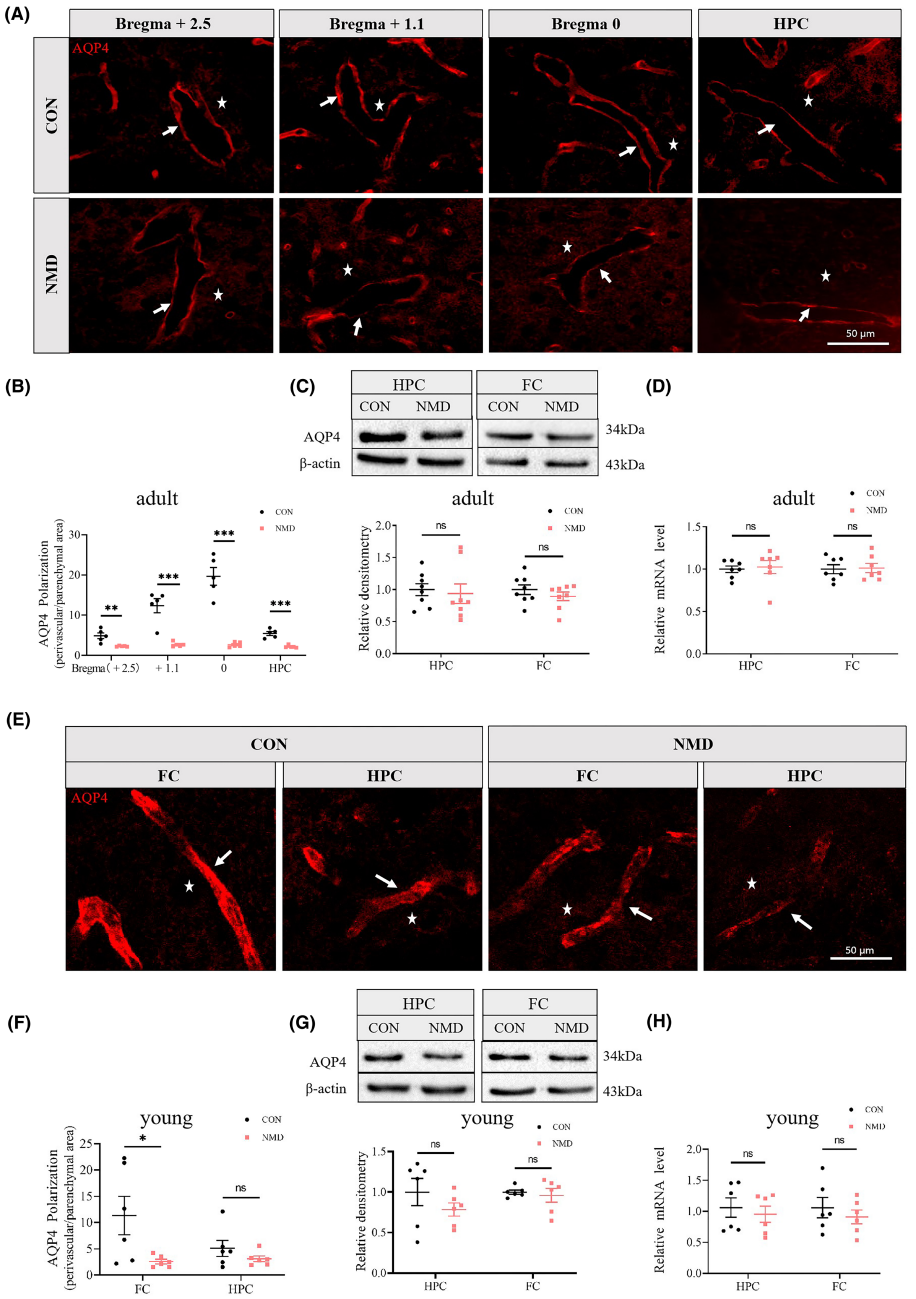
NMD reduces AQP4 polarization in the brains of young and adult mice. (D, E) Representative images of AQP4 immunostaining in the brains of adult (A) and young (E) mice, in which white arrows point to the areas around blood vessels and white stars point to parenchyma. (B, F) Quantification data showing the level of AQP4 polarization in the brains of adult (B, *n* = 5 per group) and young (F, *n* = 6 per group) mice. (C, G) WB showing the expression of AQP4 in the hippocampus (HPC) and frontal cortex (FC) of adult (C, *n* = 8 per group) and young (G, *n* = 6 per group) mice. (D, H) qPCR showing the mRNA level of AQP4 in the HPC and FC of adult (D, *n* = 7 per group) and young (H, *n* = 6 per group) mice. *p < 0.05, **p < 0.01, ***p < 0.001, ns, no significant difference, and unpaired‐sample *t*‐test.

Since glymphatic transport relies strongly on AQP4, we investigated whether the expression and polarized location of AQP4 were affected by NMD. Coronal brain sections of adult mice were sliced, and IF was performed. Immunostaining demonstrated that AQP4 expression in the CTX and HPC of the CON group was highly polarized, with a large proportion of AQP4 immunoreactivity confined to the perivascular areas. In contrast, AQP4 polarization was severely reduced in NMD mice, exhibiting a loss of polarity in astrocytic endfeet and an increase in somal labeling (Figure 4A). Quantification data showed that the ratio of AQP4 staining (perivascular space/parenchymal domains) in the CTX (Bregma +2.5, +1.1, 0 mm) and HPC (Bregma −1.1 mm) was significantly decreased in NMD mice than in CON mice (Figure 4B). This indicated that AQP4 polarization in the CTX and HPC was significantly impaired in adult NMD mice.

WB and qPCR were performed to determine whether the expression and transcription of AQP4 were influenced by NMD. WB showed no significant difference in AQP4 expression in the HPC and FC between NMD and CON mice in adulthood (Figure 4C). Consistently, qPCR showed no significant difference in the mRNA levels of AQP4 in the HPC and FC between the two adult groups (Figure 4D). This indicated that AQP4 expression in the FC and HPC of adult mice was not affected by NMD.

We next explored whether the change in AQP4 in NMD mice occurred during early life stages. Brain samples from young mice (P16) were obtained, and IF, WB, and qPCR were conducted. Immunostaining demonstrated a loss of AQP4 polarity to the astrocytic endfeet and an increase in somal labeling in the brains of young NMD mice compared with those of age‐matched CON mice (Figure 4E). Quantification of the data showed that AQP4 polarization in the FC was significantly decreased in young NMD mice than in CON mice (Figure 4F). WB showed no significant difference in the expression levels of AQP4 in the FC and HPC between the two groups (Figure 4G). Consistently, qPCR showed no significant difference in the transcription levels of AQP4 in the FC and HPC between the two groups (Figure 4H). This indicated that AQP4 polarization, rather than protein expression, in the FC was impaired by NMD in young mice. Notably, young NMD mice exhibited reduced AQP4 polarization in the HPC compared to CON mice; however, the change was not significant (Figure 4F). This indicated that NMD may have a smaller influence on AQP4 polarization in the HPC, possibly because AQP4 polarization in the HPC is almost completely established before NMD exposure.

**FIGURE 5:**
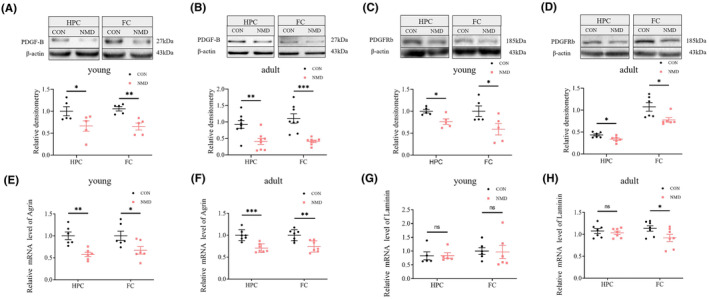
NMD decreases the expression of PDGF‐B and PDGFR‐β in the brains of young and adult mice. (A–D) WB analyses of protein extracts from the hippocampus (HPC) and frontal cortex (FC), normalized to β‐Actin level. (A, sB) WB showing the expression of PDGF‐B in the HPC and FC of young (A, *n* = 5 per group) and adult mice (B, *n* = 8 per group). (C, D) WB showing the expression of PDGFR‐β in the HPC and FC of young (C, *n* = 5 per group) and adult mice (D, *n* = 6 per group). (E–H) qPCR analysis of the mRNA level of agrin and laminin in brain tissues, expressed as the relative expression ratio normalized to the CON group. (E, F) qPCR showing the mRNA level of agrin in the HPC and FC of young (E, *n* = 6 per group) and adult mice (F, *n* = 7 per group). (G, H) qPCR showing the mRNA level of laminin in the HPC and FC of young (G, *n* = 5–6 per group) and adult mice (H, *n* = 7 per group). *p < 0.05, **p < 0.01, ***p < 0.001, ns, no significant difference, and unpaired‐sample *t* test.

Since the PDGF‐B signaling plays an important role in glymphatic system development, we next investigated whether the expression of PDGF‐B and its receptor β (PDGFRβ) was disrupted by NMD exposure. Brain tissues were harvested from young (P16) and adult mice, and WB was performed. The results showed that the expression of PDGF‐B in the HPC and FC of young NMD mice was significantly reduced compared to that of age‐matched CON mice (Figure 5A). Consistently, the expression of PDGF‐B in the HPC and FC of adult NMD mice was significantly lower than that in adult CON mice (Figure 5B). Furthermore, we found that the expression of PDGFRβ in the HPC and FC of young NMD mice was significantly lower than that in the CON mice (Figure 5C). In parallel, the expression of PDGFRβ in the HPC and FC was significantly decreased in adult NMD mice than in adult CON mice (Figure 5D).

Because extracellular matrices such as laminin and agrin secreted by pericytes are essential for AQP4 polarization, we next explored whether the transcription of laminin and agrin was affected by NMD exposure. qPCR demonstrated that the mRNA levels of agrin in the HPC and FC were dramatically reduced in young NMD mice than in peer CON mice (Figure 5E). Consistently, the mRNA levels of agrin in the HPC and FC of adult NMD mice was dramatically reduced compared to those in CON mice (Figure 5F). Moreover, the mRNA level of laminin in the FC of adult NMD mice was significantly lower than that in the CON group (Figure 5H).

### 3.3 NMD accelerated α‐syn accumulation and PD‐like symptoms in adult mice

**FIGURE 6:**
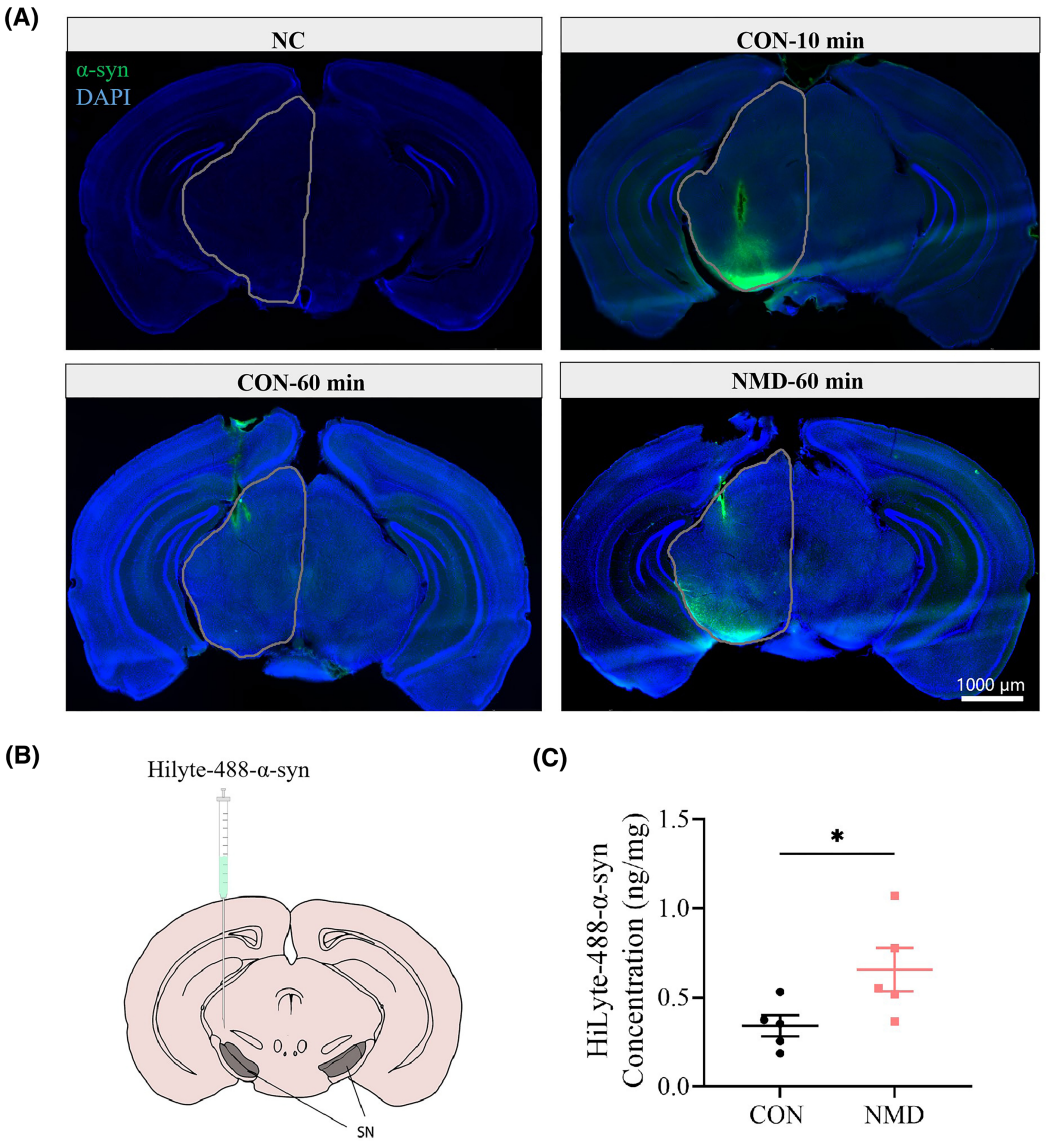
NMD impairs the clearance of injected human α‐syn from the brains of adult mice. (A) Representative images of coronal brain sections showing the distribution of HiLyte‐488‐α‐syn (green) in the brains of NMD and CON mice 10 and 60 min following injection. The white boxes depict the subcortex. NC, un‐injected control. Nuclei counterstained with DAPI (blue). (B) Schematic diagram of the intranigral injection. (C) Microplate assays demonstrating the content of α‐syn in the ipsilateral subcortex of both NMD and CON mice 60 min after injection. *n* = 5 per group. *p < 0.05, and unpaired‐sample *t*‐test.

In this section, we first explored whether the clearance of exogenous recombinant human α‐syn from the brain was impaired in adult NMD mice. HiLyte‐488‐α‐syn was slowly injected into the right SN of adult mice (Figure 6B). The residual α‐syn in the brains of mice was observed ex vivo under a microscope 10 and 60 min after injection. Representative images of coronal brain sections showed that a robust HiLyte‐488‐α‐syn signal appeared in the ipsilateral subcortex 10 min after injection into the brains of CON mice, whereas the contralateral hemisphere and the un‐injected control (NC) slice showed almost no fluorescence. The intensity of HiLyte‐488‐α‐syn declined sharply from 10 to 60 min after injection into the brains of CON mice. Representative images also displayed that more residual HiLyte‐488‐α‐syn was present in the brains of NMD mice compared with those of CON mice 60 min following injection (Figure 6A). Next, the content of HiLyte‐488‐α‐syn in the brains of mice was measured using microplate assays. The results showed that the content of α‐syn in the ipsilateral subcortex of NMD mice was significantly higher than that in CON mice (Figure 6C). This indicated that NMD could impair the clearance of α‐syn from the brain in later life, possibly owing to glymphatic dysfunction induced by NMD exposure.

**FIGURE 7:**
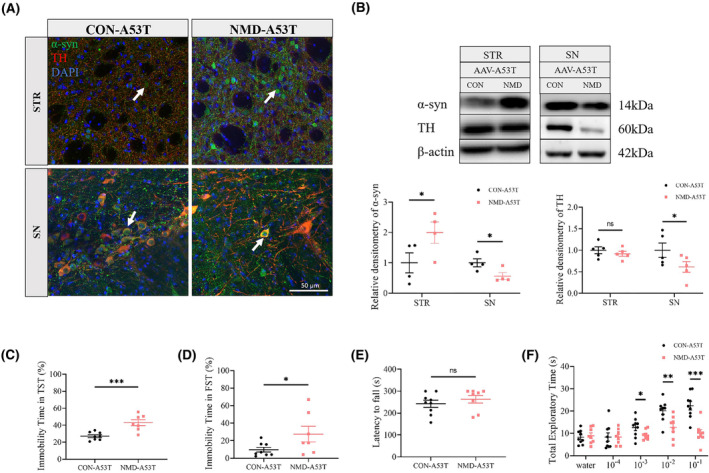
NMD accelerates pathological α‐syn accumulation and PD‐like symptoms in adult mice. (A) Immunostaining demonstrating that vector‐delivered A53T‐α‐syn (positive for Syn211 staining, green) was widely expressed in the SN and STR and co‐localized with dopaminergic neurons (positive for TH staining, red). White arrows point to intracellular α‐syn. Nuclei counterstained with DAPI (blue). (B) WB showing the level of α‐syn monomer (*n* = 4 per group) and TH (*n* = 5 per group) in the SN and STR of both groups. (C) The immobility time was measured in the TST (CON‐A53T, *n* = 8; NMD‐A53T, *n* = 7). (D) The immobility time was measured in the FST (CON‐A53T, *n* = 8; NMD‐A53T, *n* = 7). (E) The latency to fall off the rod was measured in the rotarod test (CON‐A53T, *n* = 9; NMD‐A53T, *n* = 8). (F) The total exploratory time spending on the scents was recorded in an odor discrimination test (CON‐A53T, *n* = 9; NMD‐A53T, *n* = 8). *p < 0.05, **p < 0.01, ***p < 0.001, ns, no significant difference, and unpaired‐sample *t* test.

To investigate the influence of NMD on the α‐syn accumulation and PD pathology, AAV‐A53T was bilaterally administered to the SN of 4‐week‐old NMD and CON mice. IF and WB were conducted to determine the expression of A53T‐α‐syn and TH in the brains of mice 4 weeks after transfection. Double immunostaining demonstrated that human A53T‐α‐syn (positive for Syn211 staining, green) was widely expressed in the STR and SN of both CON and NMD mice. Most dopaminergic neurons (positive for TH staining, red) co‐localized with α‐syn, indicating production of the A53T‐α‐syn transgene by dopaminergic neurons (Figure 7A). WB showed that the level of α‐syn monomer in the STR of NMD mice was significantly higher than that in the STR of CON mice (Figure 7B), indicating that more α‐syn accumulated in the brains of NMD mice. In contrast, WB showed that the level of α‐syn monomer in the SN of NMD mice was significantly lower than that in the SN of CON mice (Figure 7B). The level of TH in the SN of NMD mice was significantly lower than that in the SN of CON mice (Figure 7B). We speculated that the decreased number of dopaminergic neurons caused the reduced production of α‐syn in the SN of NMD mice.

To explore whether NMD could accelerate PD‐like symptoms, a series of behavioral tests was performed 4 weeks after AAV transfection. In both the TST and FST, the immobility time of NMD‐A53T mice was dramatically increased compared to that of the CON‐A53T group (Figure 7C,D), which was consistent with the observations in young (P16) and adult mice without AAV transfection (Figure S1B,C). In the rotarod test, there was no significant difference in the latency to fall off the rod between the two groups (Figure 7E). We speculated that NMD did not deteriorate the motor performance in 8‐week‐old mice, mostly because the motor disorder did not develop at this age. Since olfactory deficits are proposed to be prodromal for PD; hence, we next conducted an odor discrimination test in mice. In this test, NMD‐A53T mice exhibited a significant reduction in olfactory sensitivity at concentrations of 10^−3^, 10^−2^ and 10^−1^ compared to CON‐A53T mice (Figure 7F), indicating that NMD could accelerate olfactory deficits in PD model mice.

## 4 DISCUSSION

This study aimed to investigate the potential influence of ELS on the glymphatic system development and its implications in PD progression. Using DEM‐MRI, fluorescence imaging, and microplate assays, we observed that glymphatic activity was suppressed in adult NMD mice. We found that AQP4 polarization and PDGF‐B signaling were reduced in the brains of NMD mice at both young and adult age. Finally, we found that NMD accelerated the pathological accumulation of α‐syn and PD‐like symptoms in mice overexpressing A53T‐α‐syn. Our observations indicate that ELS can disrupt the development of the glymphatic system and increase the susceptibility to PD later in life.

The glymphatic system, an efficient CSF‐ISF exchange mediated by the astrocytic AQP4, serves to remove neurotoxic proteins such as Aβ, tau, and α‐syn from the brain.^1^, ^6^, ^27^, ^42^, ^43^ The age‐related evolution of this system has been investigated in elderly rodents^7^ and humans.^44^ For instance, the glymphatic activity is reported to dramatically decrease in old mice, partly owing to a decrease in arterial pulsatility and loss of AQP4 polarization.^7^ Reduced glymphatic activity with age impairs the clearance of harmful proteins from the brain, which explains why neurodegenerative diseases (e.g., PD) usually occur in the elderly. However, this does not explain why neurodegeneration occurs in some people but not in others.

Early life is a critical period of brain development. Recent studies have shown that the glymphatic system in the rodent brain develops early in life.^14^, ^15^ For instance, Munk et al. found that glymphatic flow in the mouse brain gradually increased with age. They further found that glymphatic flow displayed initial signs in the circle of Willis at embryonic period 17.5, started in the HPC at P1, and ultimately matured at P14.^15^ They also found that the progressive increase in glymphatic flow during early life was dependent on AQP4 polarization, which increases with age.^15^

The brain is highly sensitive to environmental insults early in life. Substantial evidence has shown that exposure to ELS in the form of childhood maltreatment, maternal neglect, or abuse can have short‐ or long‐term effects on brain development^16^, ^45^ and increases the risk of developing a variety of neurodegenerative and psychopathological diseases later in life.^17^, ^18^, ^19^, ^20^ However, the potential effect of ELS on glymphatic system development and PD progression is not well known. In the present study, we exposed mice to NMD for 14 consecutive days (P2‐14), a critical period for glymphatic development. We observed that NMD caused depression‐like behaviors from childhood (P16) to adulthood (Figure S1B–D), indicating that the NMD mouse model was successfully established. More importantly, we found that NMD seriously disrupted glymphatic development and increased the risk of developing PD later in life. Our results suggested that impaired glymphatic development during the neonatal period may contribute to the interindividual risk of PD later in life.

The existing literature shows that NMD can lead to immediate or permanent changes in different astrocytic biomarkers.^46^, ^47^ For instance, NMD leads to an acute reduction, followed by the reversal of GFAP expression in the mouse brain.^48^ In contrast, NMD permanently increases astrocyte‐specific specific glutamate transporters (GLT‐1, GLAST) expression in the hippocampal astrocytes of 10‐week and 18‐month‐old rats.^49^ Recently, Tanaka et al.^50^ found that NMD decreases the pericyte coverage of capillaries in the prefrontal cortex of 4‐month‐old mice. Our observations showed that AQP4 polarization in the brain was reduced in NMD mice at both young and adult age, indicating that NMD may have persistent even lifelong effects on the glymphatic system. Further studies are warranted to investigate the duration of NMD on the glymphatic system.

The influence of NMD on the development of glymphatic system in female mice remained unknown. Many studies support that male rodents are more vulnerable to NMD stress than females.^35^, ^36^ For example, it is reported that NMD induces an increase in the number of astrocytes that is only observed on males, with no changes being observed among females.^51^, ^52^ Recently, Giannetto et al.^53^ found that there is no difference in glymphatic inflow between healthy male and female mice, independent of anesthetic, time‐of‐day, brain subregions, or age. We speculate that NMD could also disturb the development of glymphatic system in females, but the extent may differ from males. Further studies are warranted to investigate the potential influence of NMD on the glymphatic system in females and the gender difference.

Preclinical studies have revealed that the AQP4 expression and glymphatic activity are severely impaired in adult mice subjected to chronic stress. For example, stressful treadmill exercise is reported to suppress the AQP4 expression partly via a moderate increase in plasma CORT.^54^ Chronic unpredictable mild stress (CUMS) downregulates AQP4 expression in the CTX and HPC of adult mice, which was speculated to account for impaired glymphatic clearance.^22^ Consistently, from our previous study, we found that chronic stress reduced the expression and polarization of AQP4 and damaged glymphatic flow in adult mice. Moreover, the administration of the glucocorticoid receptor (GR) antagonist mifepristone significantly reverses the expression and polarization of AQP4 and rescues the glymphatic activity impaired by chronic stress.^21^ However, the effects of NMD on the AQP4 mediated glymphatic system and its underlying mechanisms remain largely unknown. In this study, we found that NMD reduced AQP4 polarization in the CTX and HPC, which was consistent with the changes in the brains of adult mice exposed to chronic stress. However, the level of AQP4 expression in the brains of NMD mice remained unchanged, which differed from that observed in adult mice exposed to chronic stress.

The DAPC is essential for AQP4 polarization, and mice deficient in dystrophin, a‐syntrophin, dystroglycan, or a‐dystrobrevin exhibit reduced AQP4 polarization.^11^, ^12^, ^55^, ^56^, ^57^ Further studies showed that DAPC attach to extracellular matrix components, including laminin and agrin, which are produced by pericytes.^10^, ^11^, ^12^, ^13^, ^58^, ^59^ Aberrant pericytes can result in abnormal AQP4 polarization.^60^ PDGF‐B is essential for the development of pericytes.^61^ PDGF‐B signals via PDGFRβ, the latter is expressed by pericytes at all stages of development.^62^ This signaling is crucial for the recruitment of pericytes to the brain vasculature.^61^, ^63^ Recently, Munk et al.^15^ found that the deficiency of PDGF‐B reduces the expression of PDGFRβ and suppresses AQP4 polarization to astrocytic endfeet, indicating that pericytes and PDGF‐B signaling play a vital role in the glymphatic development. Herein, we found that the expression of PDGF‐B, PDGFRβ, and the transcription of agrin in both young and adult mice were dramatically reduced by NMD exposure, suggesting that the loss or dysfunction of pericytes. Because pericytes express GR, pericyte deficiency is possibly caused by a high level of CORT. A previous study found that incubation of primary cultured pericytes with dexamethasone induces cell apoptosis, whereas administration of mifepristone inhibits dexamethasone‐induced apoptosis.^64^ Herein, we observed that adult NMD mice exhibited high‐plasma CORT levels (Figure S1A), indicating that NMD may cause pericyte dysfunction via CORT signaling.

PDGF‐B is secreted by vascular endothelial cells.^65^ Accumulating evidence indicates that short‐term stressors induce temporary endothelial dysfunction, whereas repetitive stressors impair vascular reactivity.^66^ Specifically, NMD is reported to increase superoxide generation in the vasculature, leading to endothelial dysfunction in adult mice.^67^ Another study demonstrated a dramatic inhibition of endothelial cell proliferation in the HPC and prefrontal CTX in CORT‐treated rats.^68^ In this study, we found that NMD reduced the expression of PDGF‐B in the CTX and HPC in young and adult mice. In parallel, NMD increased basal CORT in adult mice (Figure S1A). This indicates that NMD could cause endothelial dysfunction via CORT signaling, resulting in a decrease in PDGF‐B production.

Clinically, PD is characterized by motor symptoms as well as nonmotor symptoms such as anosmia and depression.^69^ Only a small proportion (approximately 1%) suffers from familial PD, which results from genetic mutations.^70^ However, no genetic mutations have been identified in most patients with PD. These patients exhibit multiple environmental and lifestyle factors, one of which is stress.^28^ The frequency of lifetime distress is associated with an increased risk of PD. Specifically, ELS has been reported to increase the susceptibility of dopaminergic neurons to degeneration and to aggravate their susceptibility to PD.^19^, ^29^, ^30^, ^31^, ^32^ However, the underlying mechanisms are not well‐understood. Multiple studies proved that the gradual accumulation of misfolded α‐syn was implicated in the pathogenesis of PD.^24^ Recently, emerging evidence has suggested that the glymphatic system is involved in the clearance of α‐syn from the brain and that glymphatic dysfunction could accelerate α‐syn accumulation and PD progression.^6^, ^27^ In this study, we found that NMD damaged glymphatic activity (Figures 2 and 3), which was accompanied by the accumulation of α‐syn and aggravation of PD pathology (Figures 6 and 7). We speculated that NMD could reduce the glymphatic clearance of α‐syn, cause aggregation of α‐syn in the brain, and ultimately aggravate PD‐like symptoms (e.g., olfactory deficits). Moreover, multiple studies showed that NMD increased the susceptibility of dopaminergic neurons to neurotoxins (e.g., 6‐hydroxydopamine) and caused PD‐like symptoms.^19^, ^71^, ^72^, ^73^ Since glymphatic clearance is non‐selective, we speculated that NMD could reduce the glymphatic clearance of neurotoxins and increase the susceptibility of dopaminergic neurons to pathogenic PD insults. Additionally, the compromised glymphatic system caused by NMD could lead to the accumulation of other harmful proteins (e.g., Aβ and tau) in the brain and correspondingly increase the risk of other neurodegenerative diseases.

Notably, besides the glymphatic dysfunction, other mechanisms underlying the influence of ELS on PD progression also exist. For instance, it is reported that exposure to ELS increased plasma adrenocorticotropic hormone (ACTH) and CORT levels in the adult offspring, which plays a vital role in the development of neurodegenerative disease such as PD.^74^ Additionally, Ren et al.^20^ recently found that NMD induced some prodromal syndroms of PD in aging rats, and impaired the dopaminergic system in the striatum and SN. They further identified many differentially expressed genes in the striatum of NMD rats, which are enriched in the pathway of dopaminergic synapse, biological process of locomotion, and neuromuscular process controlling balance.^20^

## 5 CONCLUSION

The present study found that neonatal stress could disrupt the development of the AQP4 mediated glymphatic system through PDGF‐B signaling, thereby increasing α‐syn aggregation and susceptibility to PD in later life. These findings suggest that avoiding or alleviating neonatal stress could protect against the disruption of glymphatic development and reduce the susceptibility to neurodegeneration.

## AUTHOR CONTRIBUTIONS

LW and GX designed the research; JS, ZL, XX, JM, WZ, and SH performed the research; JS and ZL analyzed the data and constructed the figures; XX drew the schematic diagrams; LW wrote the paper.

## CONFLICT OF INTEREST STATEMENT

None declared.

## Supporting information


Figure S1.

## Data Availability

The data that support the findings of this study are available from the corresponding author upon reasonable request.
