# Supplementary figures and images for: Neonatal stress disrupts the glymphatic system development and increases the susceptibility to Parkinson's disease in later life
Source: CNS Neurosci Ther. 2024 Feb 8;30(2):e14587. doi: 10.1111/cns.14587 (PMC10851323; doi:10.1111/cns.14587)

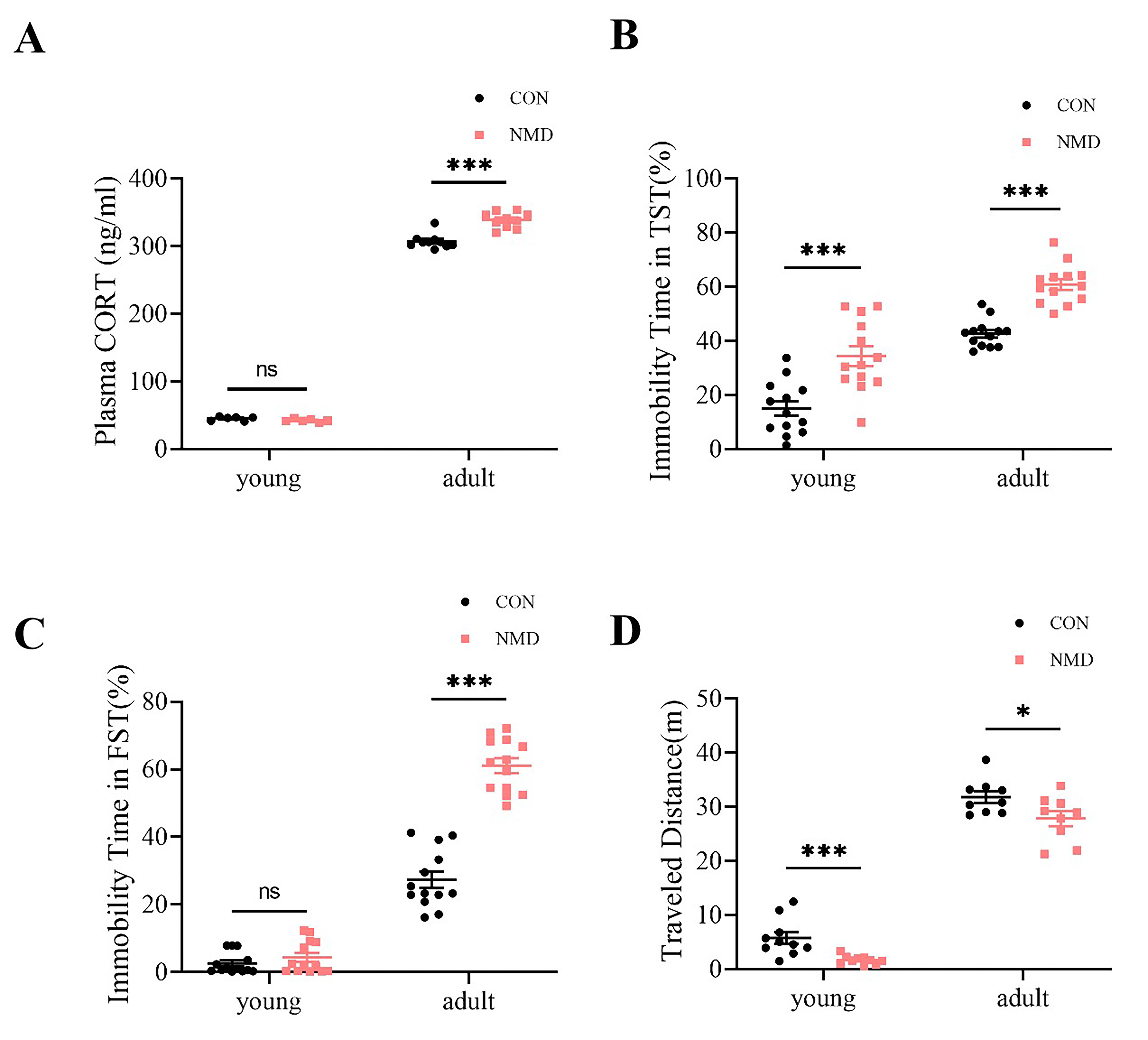

Supplement: Supplementary file 1 — Figure S1. [file CNS-30-e14587-s001.jpg]
